# Modulation of confined water dynamics in ion channels by terahertz electric fields

**DOI:** 10.1039/d5na00942a

**Published:** 2026-02-02

**Authors:** Xiaofei Zhao, Wen Ding, Hongguang Wang, Yongdong Li, Chunliang Liu

**Affiliations:** a Key Laboratory for Physical Electronics and Devices of the Ministry of Education, School of Electronic Science and Engineering, Xi'an Jiaotong University Xi'an Shaanxi 710049 China wanghg@xjtu.edu.cn

## Abstract

Water confined within nanoscale environments exhibits dynamic behaviors distinct from bulk water, with profound implications for nanodevice function. Biological ion channels, as natural nanopores, represent ideal systems for investigating such confined dynamics. The dynamical properties of water molecules in the pore regions of voltage-gated potassium and sodium channels were systematically compared, and their responses to terahertz electric fields were examined using molecular dynamics simulations. Spatial confinement and water–protein interactions markedly reduce water mobility and induce strong orientational polarization. Terahertz electric fields produce frequency-selective effects: application of a 16 THz field perpendicular to the membrane enhances water mobility, whereas a 24 THz field suppresses it, indicating selective excitation of water vibrational modes that may modulate channel function. The regulation of confined water is further shown to depend on both field strength and incident direction. These findings elucidate how terahertz fields modulate hydration dynamics, provide mechanistic insight into electric field–protein interactions, and offer guidance for the rational design of biomimetic nanofluidic systems and field-responsive biointerfaces.

## Abstract 2

Water confined in biological channels shows distinct dynamics. Terahertz fields selectively modulate this behavior, providing insight into field–protein coupling and guiding biomimetic design.

## Graphical abstract

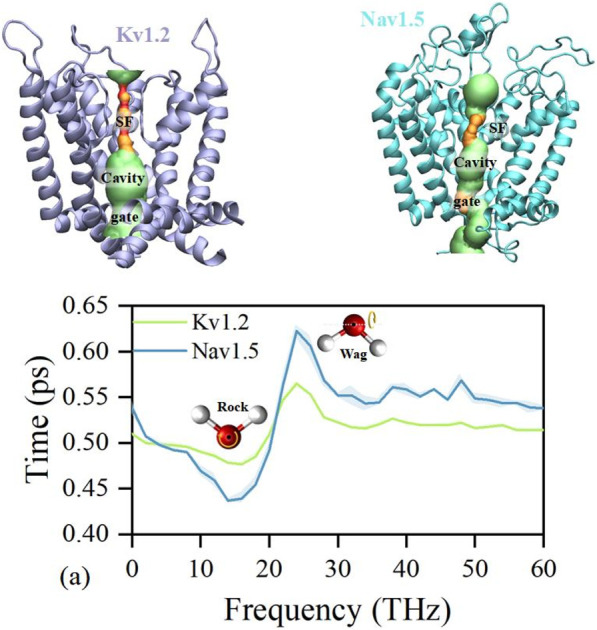

## 1. Introduction

As the most ubiquitous solvent in nature, water plays essential roles in diverse chemical, biological, and physical processes, owing to its strong polarity, hydrogen bonding, and structural flexibility.^1,2^ With the advancement of nanotechnology, it has become evident that water confined at the nanoscale exhibits markedly different behaviors compared to its bulk counterpart, including enhanced structural ordering, slowed dynamics, and reduced dielectric.^3–5^ A deeper understanding of these confined behaviors has not only advanced our knowledge of nanoscale water but also driven the development of functional devices based on nanoconfinement, laying the groundwork for novel applications in fields such as energy storage, drug delivery, and molecular separation.^6,7^

Among various nanoconfined environments, biological nanopores are molecular machines meticulously shaped by evolution and have long served as essential templates and sources of inspiration for the design of nanodevices.^8^ Confined water within these protein channels, which mediate key cellular processes, has attracted growing attention due to its critical roles in regulating transport,^9–14^ gating,^5,15–18^ conformational transitions,^19–22^ and environmental sensing.^23^ Elucidating the behavior of water in such confined biological environments is therefore essential for uncovering the physicochemical principles that govern membrane protein function.

Given the pivotal roles of water at the nanoscale in both biological systems and artificial nanofluidic devices, precisely controlling the dynamics of confined water is of fundamental importance. Such dynamics critically regulate protein structure and function,^24^ ion and molecular transport,^14,25^ energy dissipation,^26,27^ and even reaction kinetics.^28^ Elucidating and actively tuning such behaviors not only deepen our understanding of hydration phenomena but also provide a theoretical basis for the rational manipulation of cellular functions and the optimization of nanofluidic device performance. In this context, numerous studies have systematically investigated the effects of geometric and chemical characteristics of confined environments on water behavior, thereby establishing a robust theoretical framework for tailoring water dynamics *via* the rational design of nanoconfined systems.^29–31^ Beyond structural design, electric fields have attracted increasing attention in recent years as a powerful tool to modulate water dynamically. Their distinct advantages, such as real-time tunability, non-contact control, and reversibility, enable flexible and precise manipulation of water molecules. Recent *ab initio* molecular dynamics studies have examined the response of liquid water to static electric fields, revealing field-induced structural transformations as well as electric-field-activated molecular dissociation and proton transfer.^32,33^ Additionally, classical molecular dynamics simulations have examined how external electric fields modify the orientational ordering and the associated entropic contributions of bulk water, offering atomistic insight into field-induced structural organization and local entropy variations.^34^ The regulatory effects of electric fields on water molecules in confined environments have also been extensively explored. Matthew R. Powell *et al.* demonstrated that electric fields can reversibly switch carbon nanotubes between wetted and dewetted states, thereby tuning their conductive behavior.^35^ Notably, such electric-field-induced dewetting has also been implicated as a key gating mechanism in biological nanopores.^16,36^ Jianlong Kou *et al.* showed that when the electric field frequency resonates with the intrinsic hydrogen-bond vibrations, the water flux through nanotubes increases significantly.^37,38^ Zhi Zhu *et al.* reported that a 1.39 THz field disrupts hydrogen bonding within one-dimensional water chains inside carbon nanotubes, inducing super-permeation, whereas a 31.5 THz field predominantly affects hydrogen bonds in two-dimensional water layers.^39,40^ Qilin Zhang *et al.* constructed an asymmetrically hydrophilic nanoconfined system and achieved directional water transport under a 27 THz electric field, offering new insights for designing nanoscale water pumps.^25^ Collectively, these studies underscore the frequency-selective nature of electric-field modulation on confined water, with the response being highly sensitive to both the geometrical and chemical features of the confined environment. Importantly, electric fields in the terahertz range resonate with water's collective vibrational modes,^41^ positioning them as a particularly effective means of non-invasive and tunable control over water-mediated phenomena at the nanoscale.

Despite these advances, most studies have focused on inorganic nanomaterials with well-defined geometries and simple, rigid compositions. In contrast, little attention has been paid to more complex, irregular, and flexible confined environments, which often exhibit greater adaptability and functional diversity and may hold greater promise for future technological applications. Biological nanopores serve as prime examples of such environments, characterized by evolutionarily refined sizes, geometries, and surface chemistries that enable a wide range of complex biological functions. These characteristics make them ideal natural platforms for investigating the behavior of confined water molecules and their modulation by terahertz electric fields under complex and flexible confinement conditions.

In this study, molecular simulations were used to investigate the behavior of confined water molecules in two membrane proteins that play key roles in neural signal transmission, namely the voltage-gated potassium channel and the sodium channel. The study also systematically explores the regulatory effects of terahertz electric fields on the confined water molecules within these channels. The results indicate that spatial confinement and water–protein interactions significantly reduce the mobility of water molecules within the channels and induce pronounced orientational polarization. Water molecules exhibit high sensitivity to the frequency and direction of the electric field, as different frequencies excite distinct vibrational modes of the water molecules. This modulation alters water–protein interactions, thereby influencing the channel's function. This study not only deepens our understanding of the dynamics of confined water molecules in biological nanopores but also provides an essential theoretical foundation for the development of tunable biomimetic nanofluidic devices and terahertz-based biological regulation technologies.

## 2. Methods

### 2.1 Simulation model

The voltage-gated potassium and sodium channels were selected as representative models due to their critical roles in the generation and propagation of neural electrical signals. Both channels share a similar overall architecture, consisting of a voltage-sensing domain (S1–S4) and a pore domain (S5–S6) that mediates ion conduction across the membrane. The pore regions create structurally distinct confined environments for water molecules, providing an ideal platform for exploring how terahertz electric fields influence water dynamics under varying confinement conditions. The potassium channel structure was taken from the Kv1.2 channel (PDB ID: 3LUT),^42^ with the pore domain corresponding to residues 312–421. The sodium channel structure was obtained from the Nav1.5 channel (PDB ID: 7FBS),^43^ and to our knowledge remains the only resolved structure of a mammalian sodium channel in an open-state presumed conductive conformation. The pore domain includes residues 236–430, 825–945, 1320–1478, and 1643–1777. Each pore domain of the channel was inserted into a 9 nm × 9 nm POPC lipid bilayer using CHARMM-GUI.^44^ The systems were then solvated with KCl or NaCl solutions to ensure electrostatic neutrality. In the potassium channel model, the aqueous layers on both sides of the membrane were approximately 2 nm thick, containing a total of 12 409 water molecules. In contrast, the sodium channel model featured an expanded extracellular water layer of about 4 nm to accommodate its larger extracellular domain and ensure sufficient protein hydration, containing a total of 22 424 water molecules. The corresponding molecular dynamics models are shown in SI Fig. S1. After system construction, energy minimization and stepwise equilibration were performed using GROMACS.^45^ The equilibration followed the multi-stage protocol recommended by CHARMM-GUI, with extended durations at each stage, resulting in a total equilibration time of 93 ns. During this process, restraints on the protein and lipid molecules were gradually reduced. In the subsequent production simulations, all restraints were entirely removed.

### 2.2 Simulation setup and data analysis

All simulations in this study were performed using the CHARMM36 force field^46^ and the TIP3P water model within GROMACS 2019.4. TIP3P is widely used in protein simulations due to its reliable description of biomolecular behavior within the CHARMM force field framework.^47–49^ However, it does not account for electronic polarization or quantum effects,^33,50^ which may contribute to anharmonic responses of water molecules, particularly under strong external electric fields. More accurate approaches, such as *ab initio* molecular dynamics, can capture these effects but are computationally prohibitive for the large membrane–protein systems studied here. Moreover, the applied electric field strengths in this study correspond to only a few percent of the system's intrinsic field strength.^51–53^ Under these conditions, the use of a non-polarizable water model is a reasonable choice for describing the main dynamical behavior of water and protein while maintaining computational feasibility. Molecular structures and simulation results were visualized using the Visual Molecular Dynamics (VMD) software.^54^ Electrostatic interactions were calculated using the Particle Mesh Ewald (PME) method^55^ to accurately account for long-range electrostatics, with a real-space cutoff distance set at 1.2 nm. Van der Waals interactions were handled using a cutoff scheme, applying force-switch smoothing between 1.0 nm and 1.2 nm to ensure a smooth decay near the cutoff. The system temperature was maintained at 310 K using the velocity-rescaling thermostat (V-rescale).^56^ The simulation trajectories were recorded at intervals of 0.1 ps, yielding a total of 10^6^ frames per simulation. Each set of conditions was simulated in triplicate, and the results were averaged over the three independent runs for subsequent analysis.

The electric field applied in this study is described by 
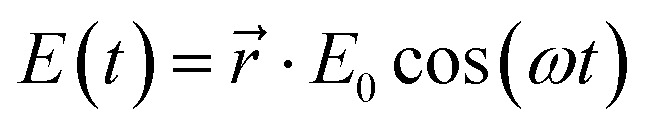
, where *E*_0_ represents the field strength and 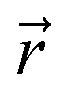 denotes the direction of the field. Unless otherwise specified, the field strength *E*_0_ is set to 0.4 V nm^−1^ and applied along the channel axis (*z*-direction). This strength is consistent with the magnitude of local electric fields commonly present in biomolecular systems, which are typically on the order of V nm^−1^ and can reach up to 10 V nm^−1^ in bulk water.^57–59^ The parameter *ω* denotes the angular frequency of the electric field. For each channel, 97 distinct field parameter combinations were systematically investigated to examine their influence on the dynamics of confined water.

The pore radius profiles of the ion channels were calculated using the HOLE program^60^ and visualized with VMD to assess the geometric features of the confined regions. The spatial variation of water residence time was evaluated by averaging the duration that water molecules remained within consecutive 0.1 nm slices along the *z*-axis. To assess the effect of the electric field on the residence time of confined water molecules, water within the selectivity filter (SF) and cavity was analyzed in the sodium channel. In contrast, due to the highly restricted nature of the SF region in the potassium channel, where water exchange is negligible, only the cavity was considered. Additional structural and dynamical properties, including the number of hydrogen bonds, solvent-accessible volume, and radial distribution functions, were evaluated using the built-in analysis tools of the GROMACS software suite. Hydrogen bonds were identified using standard geometric criteria, with a donor to acceptor distance of ≤0.35 nm and an angle of ≤30°formed by the hydrogen, donor, and acceptor atoms. Donor and acceptor atoms were assigned automatically based on the system topology, as implemented in the gmx hbond tool. The interaction energy between protein residues and water molecules was analyzed using classical molecular mechanics potentials, which include coulombic and van der Waals contributions. The coulombic interaction was calculated as
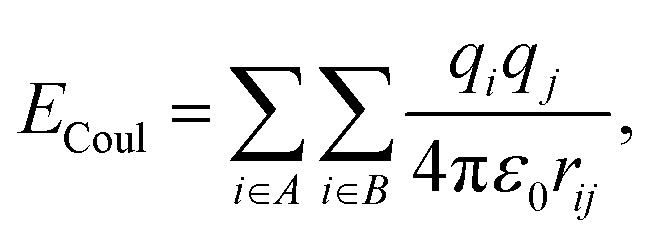
and the van der Waals interaction was modeled using the 12-6 Lennard-Jones potential:
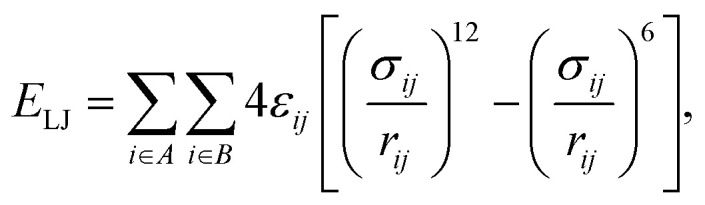
where *A* and *B* represent the sets of selected protein residues and water molecules, respectively; *q*_*i*_ and *q*_*j*_ are the atomic charges; *r*_*ij*_ is the distance between atoms *i* and *j*; and *ε*_*ij*_ and *σ*_*ij*_ are the Lennard-Jones parameters for the atom pair (*i*, *j*). All parameters are derived from the employed force field.

## 3. Results and discussion

### 3.1 Comparison of water confined in Kv and Nav channels

**Fig. 1:**
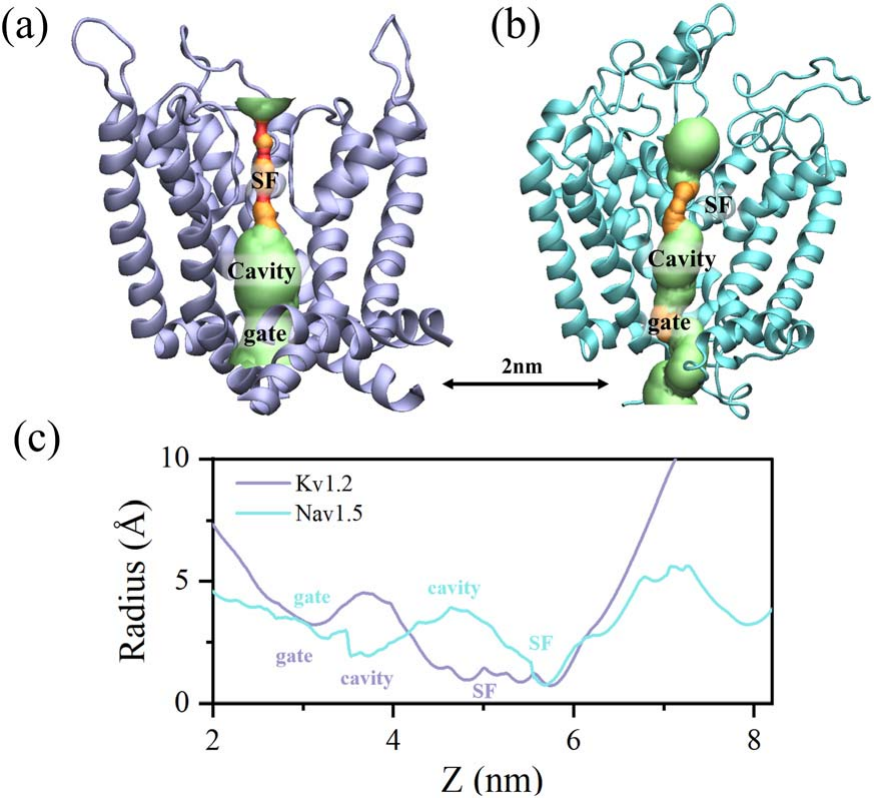
Comparison of pore structures in ion channels. (a) Pore region of the potassium channel. (b) Pore region of the sodium channel. The position *z* = 2 nm is marked in panels (a and b) to indicate the starting point corresponding to panel (c). (c) Comparison of pore radius values.

The pore radius profiles of the potassium and sodium channels, calculated using the HOLE program, are presented in Fig. 1. In these profiles, different radii are represented by distinct colors, with red indicating radii less than 1.15 Å, orange corresponding to 1.15–2.3 Å, and green denoting radii greater than 2.3 Å. Both channels generate pronounced spatial confinement for water molecules within the cavity and SF regions; however, their pore geometries differ substantially. The potassium channel displays a relatively straight and regular architecture, with a narrow and elongated SF approximately 1.2 nm in length, where the minimum radius is about 0.15 nm, thereby restricting water permeation to a single-file arrangement. In contrast, the sodium channel exhibits irregularities and distortions within its pore, with a broader yet shorter SF. The constriction of comparable minimal radius is confined to only a narrow region, while the cavity is considerably narrower, a feature that may promote dehydration and facilitate the transition to an inactivated state. Furthermore, the sodium channel possesses a substantially larger extracellular vestibule, within which an additional confined region is evident. To assess whether the channels remained hydrated or experienced dewetting during the simulations, water density distributions along the pore axis were calculated and are presented in Fig. S2. The results confirm that both channels maintained a hydrated state under the simulated conditions, providing a necessary foundation for subsequent analysis of confined water dynamics.

**Fig. 2:**
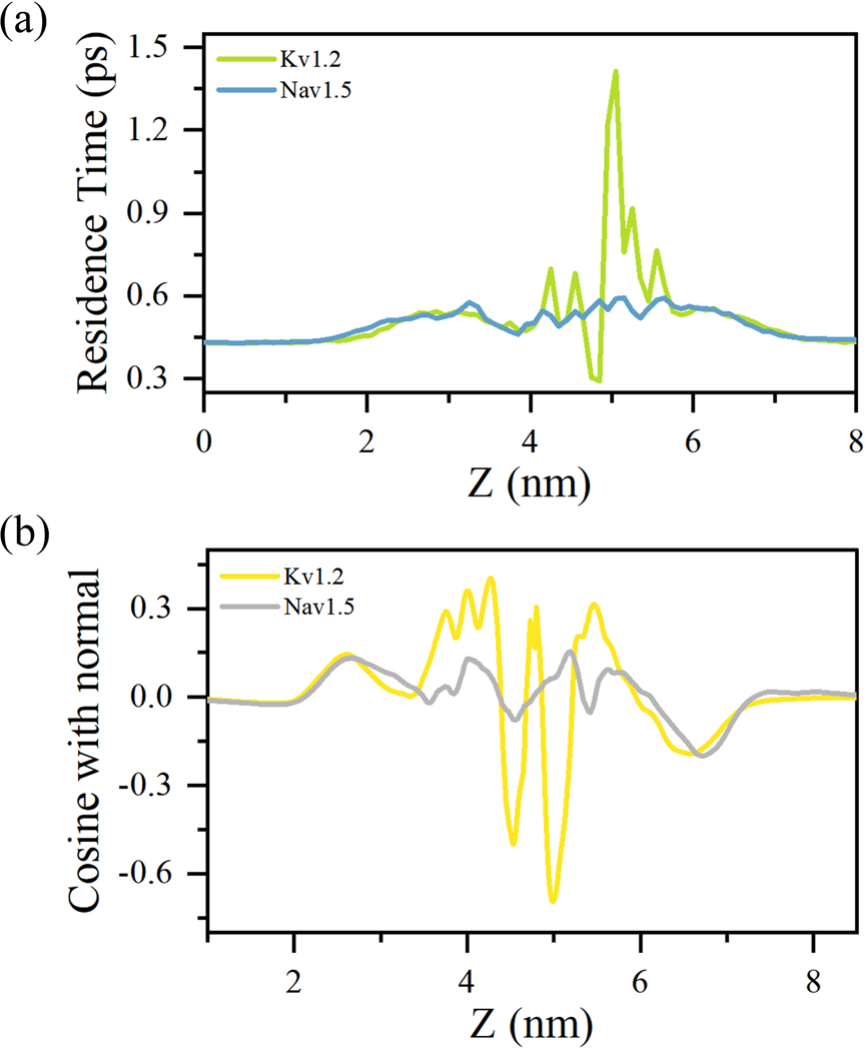
Properties of confined water molecules in the channel pore. (a) Variation of water residence time along the *z*-direction within the pore region. (b) Dipole orientation of water molecules as a function of the *z*-coordinate.

According to the structural analysis presented above, water molecules are confined within the pore regions of ion channels, resulting in dynamical behaviors distinct from those in bulk water. The residence time distributions of water molecules along the pore axis (*z*-direction) in both potassium and sodium channels are shown in Fig. 2a. In both systems, the average residence time in the bulk region is approximately 0.43 ps, with no significant difference between the channels. As the pore narrows, the residence time increases and reaches a maximum of about 0.54 ps in the gate region, where the constriction is most pronounced. The sodium channel displays an even longer residence time in this region, likely reflecting its narrower gate. Beyond the gate, the pore widens and the residence time decreases accordingly. Approaching the SF, a secondary increase emerges, which can be attributed to electrostatic interactions with ions and polar residues located in this region. In the cavity regions of both channels, the dynamics of water molecules are generally similar, with no substantial differences observed. In the SF regions, however, their behavior diverges markedly. In the potassium channel, water molecules adopt a single-file configuration with highly restricted mobility, resulting in an average residence time longer than 1 ns, which is considerably greater than in the cavity. By contrast, the voltage-gated sodium channel accommodates more flexible ion–water coordination, allowing water molecules to move around sodium ions within the SF. Consequently, the residence time in the SF region remains comparable to that in the cavity. These contrasting dynamic features highlight the fundamentally different hydration and transport mechanisms between potassium and sodium channels. To provide a more intuitive understanding, the contrasting dynamic behaviors of water molecules within the SF regions of the two channels are visualized in the SI Video.

Moreover, as illustrated in Fig. 2b, compared to the randomly oriented dipoles of water molecules in the bulk solution, the spatial confinement and distinctive electrostatic environment within the channel pore significantly promote dipole alignment. In both potassium and sodium channels, water molecules below the gate exhibit comparable polarization, reaching a maximum near the gate region where dipole vectors form an angle of roughly 80° relative to the channel axis. Beyond this point, the alignment progressively diminishes. The narrower cavity of the sodium channel induces slightly stronger polarization than that of the potassium channel. In the SF, however, pronounced differences arise, reflecting distinct residue composition, pore geometry, and ion distribution. The sodium channel maintains a level of dipole polarization in the SF comparable to that of its cavity. In contrast, in the potassium channel, water molecules demonstrate markedly enhanced polarization within the SF, where they adopt a single-file configuration under more stringent spatial confinement. These findings highlight the sensitivity of water behavior in confined environments to local structural and physicochemical determinants.

### 3.2 Terahertz electric field effects on water behavior in channel pores

The preceding analysis indicates that water molecules confined within potassium and sodium channels exhibit dynamic behaviors markedly distinct from those in the bulk phase, primarily due to pronounced spatial restriction and the distinctive electrostatic environment within the pore. Such confined water exhibits pronounced sensitivity to its local environment, suggesting that external perturbations could substantially influence its behavior. Given the strong sensitivity of water molecules to terahertz fields, the regulatory effects of terahertz electric fields of varying frequencies on confined water dynamics were examined. The structural stability of the channels under terahertz fields, assessed through root-mean-square deviation and solvent-accessible volume as presented in Fig. S3 and S4, showed negligible changes with no discernible frequency dependence. These findings indicate that terahertz fields exert minimal influence on the channel structure, implying that the observed modulation of water dynamics arises primarily from direct interactions between the fields and the confined water molecules rather than from structural alterations of the channels themselves.

**Fig. 3:**
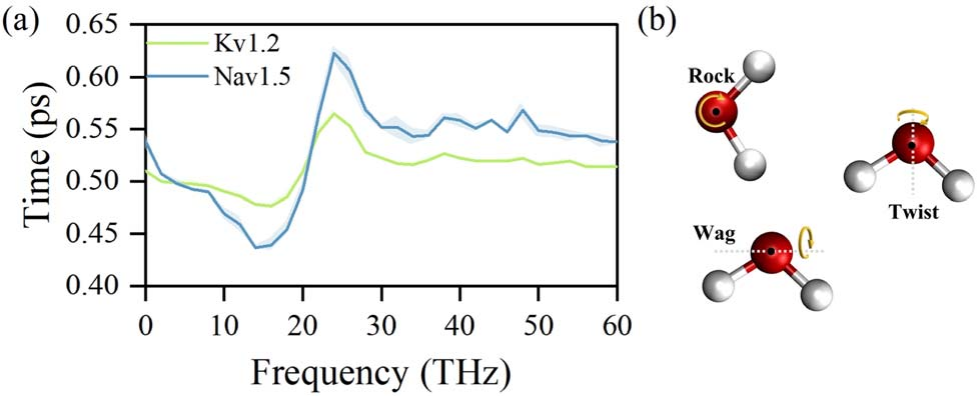
Effects of terahertz electric fields on water dynamics within ion channel pores. (a) Frequency-dependent changes in the average residence time of water molecules under terahertz electric fields. (b) Schematic representation of the three intermolecular librational modes of water: rock, twist, and wag.

The influence of terahertz electric fields at different frequencies on the dynamics of water molecules confined within the channel pore was subsequently evaluated. To quantify these effects, the average residence time of water molecules within 0.1 nm-thick slabs along the pore axis was calculated, as shown in Fig. 3a. Detailed computational procedures are provided in the Methods section. The results indicate that the dynamics of water molecules confined within the channel pores exhibit a strong dependence on the frequency of the applied electric field, showing consistent patterns across both potassium and sodium channels. In the frequency range of 0 to 16 THz, the average residence time of water molecules decreases with increasing frequency, indicating that terahertz fields in this regime enhance axial water transport, with the minimum residence time occurring near 16 THz. In contrast, between 16 and 24 THz, the residence time increases with frequency and reaches a pronounced maximum at approximately 24 THz, suggesting reduced water mobility under these conditions. At frequencies exceeding 24 THz, the residence time decreases again and gradually approaches the baseline observed under field-free conditions, indicating a diminished influence of the electric field at higher frequencies. This non-monotonic frequency dependence implies that water molecules may undergo resonant coupling with the terahertz field at specific frequencies, leading to modulation of their transport behavior in confined environments. From the above analysis, 16 THz and 24 THz emerge as two representative frequencies that strongly influence water dynamics in confined pores. Fig. S5 further illustrates the *z*-axis residence time profiles of water molecules under electric fields at these frequencies, providing a more intuitive visualization. Due to the high confinement of water molecules within the SF of potassium channels, the effect of the field is limited in this region. In contrast, field-induced modulation is more pronounced in the cavity region, while water molecules near the gate region experience a reduced effect due to their proximity to the bulk solution. In sodium channels, where the SF is wider than in potassium channels, water molecules can exchange more freely, and the electric field exerts a significant influence on the SF and cavity regions, though the effect diminishes near the gate. Furthermore, an additional extracellularly confined region in the sodium channel also exhibits substantial field-induced modulation, particularly under the 16 THz field.

To further clarify the interaction, the coupling between water molecular motions and the applied field frequencies was analyzed using a harmonic oscillator model. The harmonic oscillator model *E*_b_ = 1/2*I*(2π*f*)^2^ provides a quantitative relationship among the binding energy *E*_b_, moment of inertia *I*, and vibrational frequency *f*, thereby allowing the estimation of mode-specific frequencies based on the molecular moment of inertia and the corresponding binding energy.^61^ The binding energy of the water molecule is taken as 19 kcal mol^−1^.^62^ Based on the TIP3P water model, the moments of inertia associated with the three intermolecular librational modes, rock, twist, and wag, are 1.7709, 1.1557, and 0.6151 amu Å^2^, respectively.^46^ These characteristic vibrational modes are illustrated in Fig. 3b. The rock mode involves in-plane oscillation about an axis perpendicular to the molecular plane and passing through the center of mass. In the twist mode, the two hydrogen atoms move out of the molecular plane in opposite directions. In contrast, in the wag mode, both hydrogen atoms move in the same direction perpendicular to the plane. Based on these moments of inertia and the harmonic equation, the corresponding vibrational frequencies are estimated to be approximately 15 THz for rock, 18.7 THz for twist, and 25.6 THz for wag. Experimental spectra show that water librational motions are primarily distributed between 10 and 30 THz,^63–65^ with the rock mode centered near 15 THz and frequencies above ∼20 THz predominantly associated with wag mode.^66^ These spectral characteristics are consistent with the frequency estimates obtained in this study using the harmonic oscillator model.

Notably, the frequency of the rock librational mode closely matches the 16 THz field, which most effectively enhances water transport. In contrast, the wag mode aligns with the 24 THz field, corresponding to the most substantial suppressive effect. These observations indicate that the frequency-dependent modulation of confined water dynamics is primarily governed by selective excitation of specific librational modes by terahertz electric fields. The 16 THz field predominantly excites the rock mode, characterized by angular displacements of water molecules, thereby increasing their rotational freedom and facilitating reorientation within the confined pore. This activation contributes to the observed acceleration of water movement along the channel axis. Conversely, the 24 THz field primarily activates the wag mode, inducing localized motions that impede axial movement. At higher frequencies, the capacity of water molecules to respond effectively to the oscillating field diminishes, likely due to constraints in rotational inertia and relaxation timescales, leading to a gradual attenuation of the modulation effect. These findings underscore the critical role of vibrational mode selectivity in mediating field–molecule interactions under nanoscale confinement.

Previous studies by other researchers have investigated the modulation of water behavior by terahertz electric fields in systems such as carbon nanotubes and graphene nanoslits.^37,61^ However, the frequency-dependent effects observed in those studies differ markedly from those identified in the present work. This discrepancy underscores the critical role of the characteristics of confined environments in shaping the response of water molecules to terahertz fields. Within specific confined environments, certain vibrational modes of water may be selectively excited, resulting in diverse and system-specific transport dynamics. These findings highlight the unique value of biological channels, with their structural complexity and functional diversity, as model systems for investigating confined water dynamics and exploring strategies for external modulation.

**Fig. 4:**
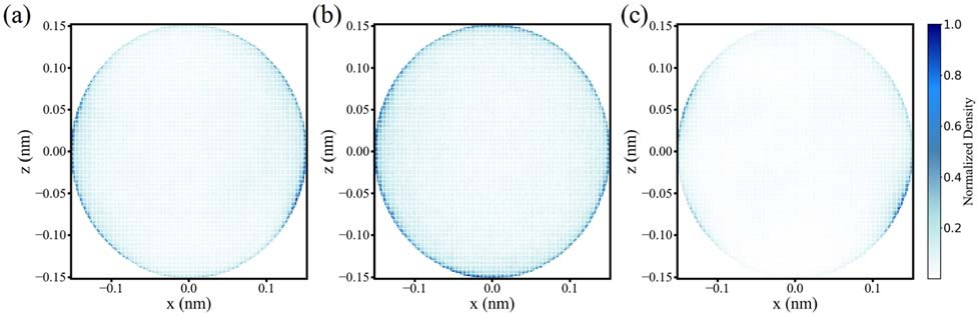
Influence of terahertz electric fields on the rotational orientation of water molecules. (a) Distribution of H–H vectors of water molecules in the potassium channel on the *x*–*z* plane under field-free conditions. (b) Distribution of H–H vectors under a 16 THz electric field. (c) Distribution of H–H vectors under a 24 THz electric field.

To further investigate the selective excitation of distinct vibrational modes of water molecules by terahertz electric fields, the distributions of H–H vector components of water molecules within the pore region were analyzed. The H–H vector of each molecule was determined by tracking the coordinates of water molecules in the cavity and computing the vector between the two hydrogen atoms, which was then decomposed into components along the Cartesian axes. For the potassium channel, the components of the H–H vector along the pore axis are shown in Fig. 4. Under field-free conditions, the distribution is relatively concentrated, indicating limited rotational freedom. Exposure to a 16 THz electric field broadens this distribution, indicative of enhanced rotational fluctuations and increased molecular reorientation, consistent with the excitation of the rock librational mode. Conversely, at 24 THz, the H–H vector distribution becomes markedly more concentrated, suggesting constrained rotational freedom corresponding to the dominance of the wag librational mode.

Water molecules within the sodium channel display a similar frequency-dependent behavior, as presented in Fig. S6. These results demonstrate that terahertz electric fields selectively modulate the rotational dynamics of confined water molecules in a frequency-dependent manner, highlighting the distinct excitation of different librational modes.

### 3.3 Effects of terahertz electric fields on water–channel interactions

**Fig. 5:**
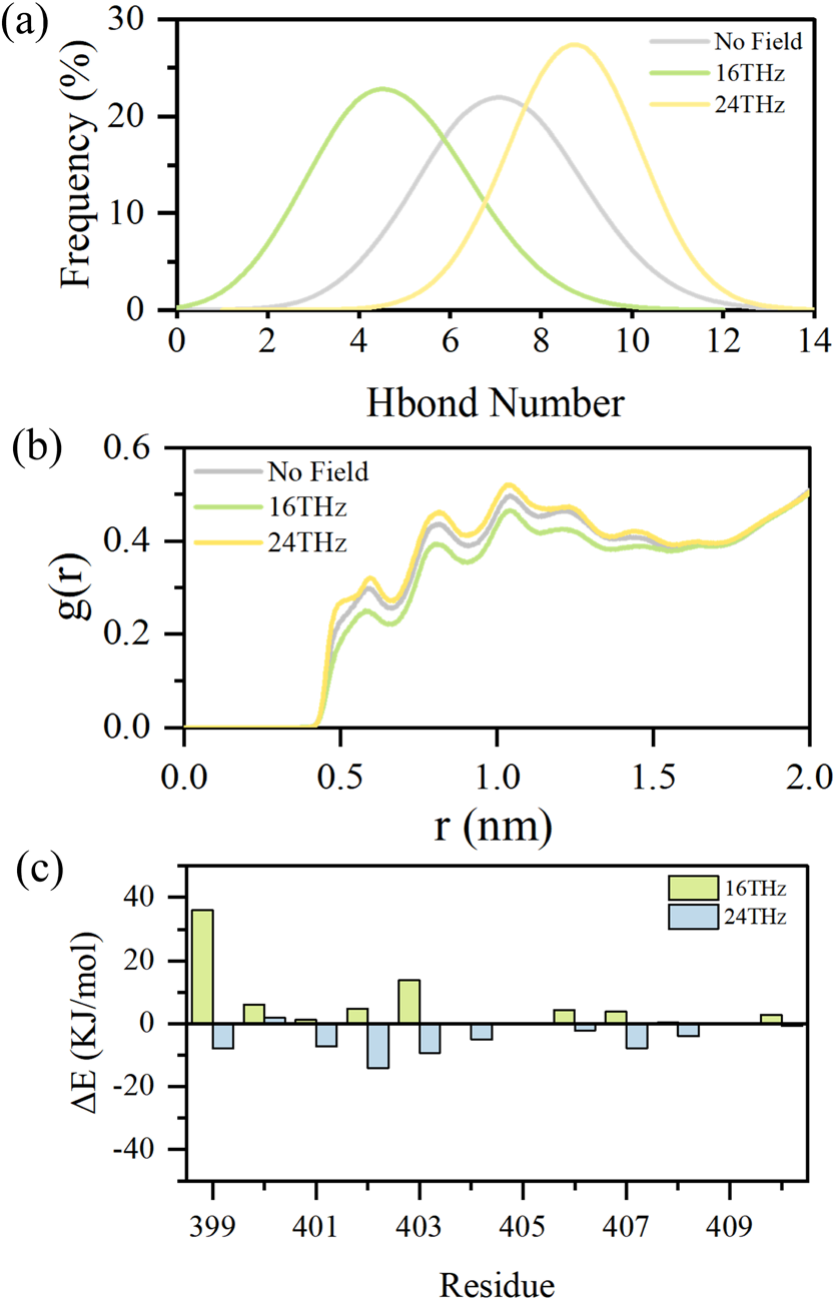
Effects of terahertz electric fields on the interactions between water molecules and the potassium channel. (a) Distribution of the number of hydrogen bonds formed between water molecules and residues within the channel pore; (b) radial distribution function (RDF) of water molecules in the pore region with reference to residue VAL406; (c) interaction energies between water molecules and residues within the pore.

The above analysis suggests that terahertz electric fields selectively excite specific vibrational modes of water molecules within the channel, thereby enhancing or restricting their dynamic behavior. Alterations in the vibrational states of water molecules may further affect their interactions with residues lining the channel, ultimately modulating the channel function. To elucidate this mechanism, the hydrogen bond formation between water molecules and pore region residues of both potassium and sodium channels was quantified. The results are presented in Fig. 5a and S7, respectively. Exposure to terahertz electric fields induces frequency-dependent modulation of hydrogen bonding between the channel and the surrounding water molecules. In the potassium channel, a 16 THz field reduces the average number of hydrogen bonds by 33.5%, whereas a 24 THz field increases hydrogen bonding by 22.9%. These findings indicate that the 16 THz field weakens the interactions between water molecules and the protein, leading to less stable binding. In comparison, the 24 THz field strengthens these interactions, resulting in enhanced binding stability.

To further substantiate this regulatory effect, the spatial organization of water molecules was evaluated using the radial distribution function relative to the gating residue 406VAL of the potassium channel. This analysis, presented in Fig. 5b, characterizes the probability of locating water molecules at varying distances and thus captures changes in their local arrangement and interaction strength. Under the 16 THz field, water molecules were more loosely distributed, whereas under the 24 THz field, they became more tightly packed. These findings corroborate the hydrogen bond analysis, confirming that the 16 THz field disrupts water–channel binding, while the 24 THz field enhances binding stability.

Building on the hydrogen bond and RDF analyses, the energetic consequences of terahertz electric fields were further evaluated through calculations of residue–water interaction energies within the channel region. The interaction energy values represent the differences between systems under an applied electric field and field-free conditions as presented in Fig. 5c. Positive values indicate that the applied field destabilizes binding interactions, whereas negative values suggest enhanced stability. Under a 16 THz electric field, interaction energies between water molecules and channel residues generally increased, with the energy involving 399VAL exhibiting the most significant rise of approximately 36 kJ mol^−1^, indicating that this frequency promotes destabilization of binding. In contrast, under a 24 THz electric field, interaction energies decreased overall, with the energy involving 402ILE showing the most significant reduction of about 14 kJ mol^−1^, suggesting that this frequency enhances binding stability. RDF analysis indicates that these energy changes likely result from alterations in the spatial distribution of water molecules around the residues, prompting a quantification of water molecules within 0.5 nm of each residue to assess the effect of terahertz fields, as shown in Fig. S8. Under the 16 THz field, the local water population decreases, weakening the cumulative coulombic and van der Waals interactions with the residue and leading to higher, less favorable interaction energies. In contrast, the 24 THz field increases the local water density, enhancing these interactions and producing more negative, energetically favorable values. This mechanistic relationship accounts for the observed changes in interaction energies. Fig. S9 further shows that the sodium channel exhibits a similar trend, reinforcing the generality of the observed pattern.

The above analysis indicates that the local dynamic behavior of water molecules in confined regions undergoes significant changes, which in turn alter their interactions with the surrounding protein environment. By modulating water–channel interactions, these changes may influence essential kinetic processes such as the exchange dynamics of water between the pore and the bulk solution, ultimately impacting channel permeability.^67^ The permeability is a fundamental characteristic that directly underlies the generation and propagation of physiological electrical signals.^68^

**Fig. 6:**
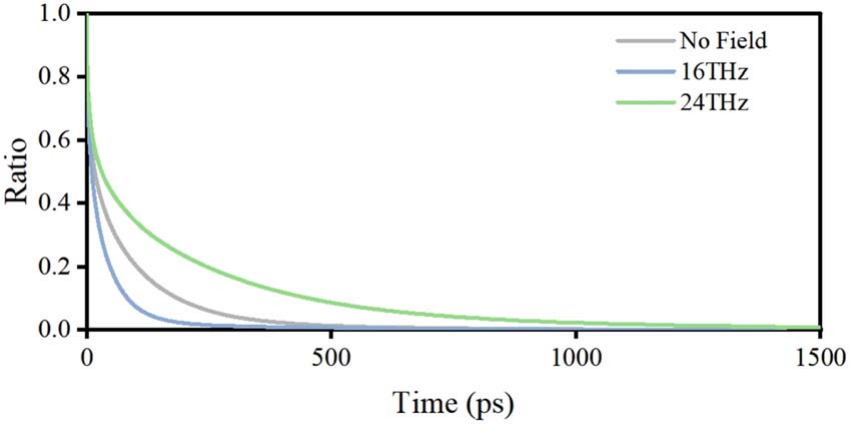
Terahertz field-induced modulation of water exchange dynamics in the confined region of the potassium channel.

To further examine this hypothesis, the exchange dynamics of water molecules between the confined region of the potassium channel and the bulk solution were analyzed, as presented in Fig. 6. The average residence time of water molecules was estimated by using the gating residue 406VAL as a reference and tracking the retention ratio of water molecules within a 1 nm radius over time. The results indicate that a 16 THz electric field markedly enhances the exchange rate of water molecules, reducing the average residence time by 47.4% compared to field-free conditions. In contrast, a 24 THz electric field significantly suppresses water exchange, leading to an average residence time 2.36 times longer than that under field-free conditions. A similar regulatory effect was observed in the sodium channel, as presented in Fig. S10. These findings suggest that excitation of the rock vibrational mode promotes the rotational rearrangement of water molecules, facilitating their migration within the pore region. In contrast, excitation of the wag mode induces a local structural stabilization, reducing the mobility and exchange of confined water molecules.

In summary, terahertz electric fields can modulate the microscopic dynamics of water molecules, thereby altering their interactions with channel proteins and influencing the channel process. These results provide mechanistic insights into how external electric fields can be harnessed to regulate biological processes precisely.

### 3.4 Parameter-dependent effects of terahertz electric fields on confined water molecules in ion channels

The preceding analysis demonstrates that terahertz electric fields exert frequency-dependent effects on the behavior of water molecules within ion channels. This modulation influences both the dynamics of confined water molecules and their interactions with the channel protein, potentially affecting the overall ion transport kinetics. In the following section, the effects of additional field parameters, including incident direction and field strength, on water behavior within the confined pore region are further examined. Based on the pronounced effects observed at 16 THz and 24 THz, these two frequencies were selected as representative cases for subsequent analysis.

**Fig. 7:**
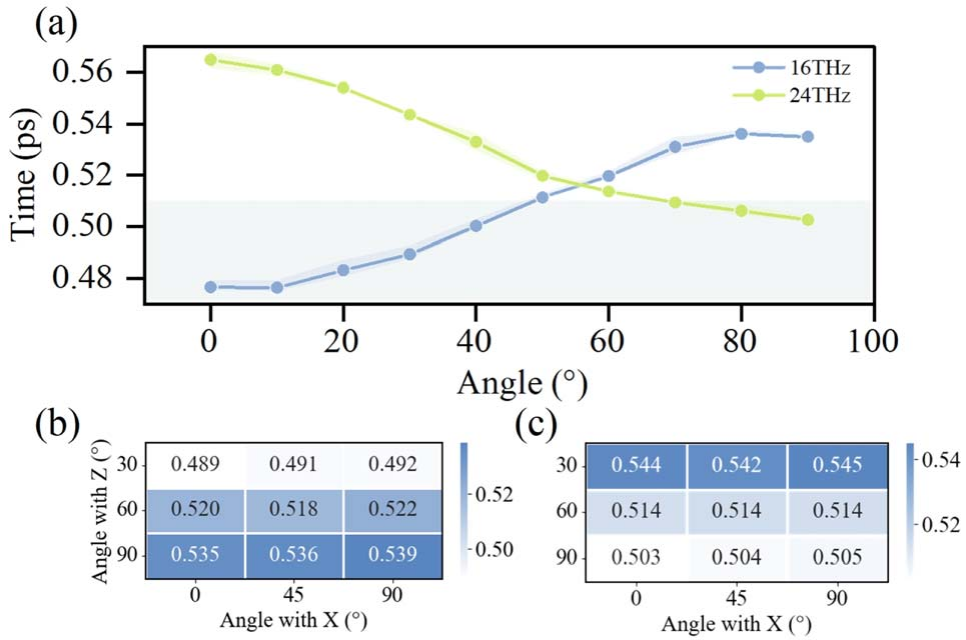
Directional dependence of terahertz electric field effects on water dynamics within the pore region of the potassium channel. (a) Modulation of water molecule average residence time by electric fields with varying angles relative to the *z*-axis in the *xz* plane. (b) Directional dependence of water dynamics under a 16 THz electric field. (c) Directional dependence of water dynamics under a 24 THz electric field.

Water molecules within the pore domain exhibit pronounced orientation polarization, as shown in Fig. 2b, suggesting that variations in the incident direction of the terahertz electric field may differentially influence their behavior. To examine this possibility, the effects of 16 THz and 24 THz electric fields applied at varying incident angles relative to the *z*-axis on the dynamics of confined water molecules were analyzed. The field directions were defined with respect to the Cartesian coordinate system, with its orientation illustrated in Fig. S1. The directional responses of water molecules in the potassium and sodium channels are presented in Fig. 7a and S11a, respectively. Despite minor differences, both channels display a similar dependence on the orientation of the applied electric field. A notable reversal in the field-induced modulation of water dynamics was observed when the incident angle of the terahertz electric field relative to the *z*-axis approached 60°. At this critical angle, the 16 THz field transitions from enhancing to suppressing water mobility, whereas the 24 THz field exhibits the opposite behavior. Notably, the magnitude of enhancement and suppression induced by the 16 THz field is comparable, while the enhancement effect of the 24 THz field applied parallel to the membrane is considerably weaker than the suppression effect observed when it is applied perpendicular to the membrane. This observation further supports the notion that 16 THz and 24 THz electric fields excite distinct vibrational modes of water molecules. These modes produce orientation torques that vary in both magnitude and direction, resulting in differential sensitivity of water molecules to the incident direction of the applied field. Furthermore, the directional dependence observed here provides strong evidence that the impact of the terahertz field on water dynamics arises predominantly from non-thermal mechanisms. If the effect was purely thermal, such pronounced directional dependence would not be expected, as also noted by Chen Song *et al.* in their study.^69^

To further examine the influence of electric field orientation on the dynamics of confined water molecules, terahertz fields were applied at angles of 30°, 60°, and 90° relative to the *z*-axis. For each case, the field direction within the xy-plane was additionally varied at 0°, 45°, and 90° relative to the *x*-axis. The corresponding results are shown in Fig. 7b, c and S11b, c. The analysis reveals that the water molecules' response is primarily governed by the angle with respect to the *z*-axis, with negligible sensitivity to in-plane variations. The pronounced directional selectivity can be attributed to the axially ordered alignment of water molecules within the channel pore.

**Fig. 8:**
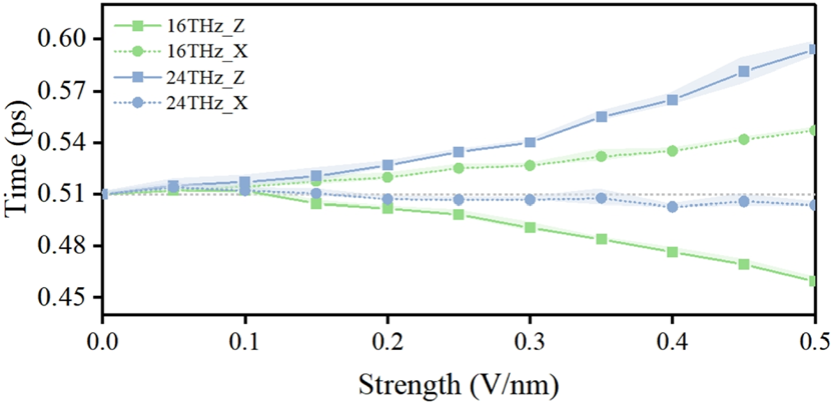
Field-strength dependence of terahertz electric field effects on water molecules in the potassium channel.

In addition, the regulatory effects of terahertz electric fields on biomolecules are strongly dependent on field strength. Accordingly, the responses of confined water molecules within the channel to electric fields of varying strengths were analyzed. The corresponding results are presented in Fig. 8 and S12. The results indicate that when the electric field strength exceeds approximately 0.1 V nm^−1^, the dynamical behavior of water molecules begins to exhibit noticeable changes, and the modulation effect becomes progressively more pronounced with increasing field strength. Notably, the 24 THz field applied parallel to the membrane plane shows markedly reduced sensitivity to field strength compared to other configurations. This observation is consistent with previous studies, which suggest that field strengths on the order of 0.1 V nm^−1^ are generally required to induce discernible effects within the limited timescales accessible to molecular dynamics simulations.^70^ Consequently, most published studies investigating electric field–protein coupling phenomena employ field strengths exceeding 0.1 V nm^−1^.^71^

This observation further suggests that terahertz fields at different frequencies may excite distinct vibration modes of water molecules, and these modes exhibit significantly different dependencies on field strength. Moreover, the incident direction of the electric field influences the excitation efficiency of these modes, revealing a coupled dependence of the terahertz modulation mechanism on both frequency and direction.

## 4. Conclusion

This study, based on molecular dynamics simulations, systematically investigates the regulatory effects of terahertz electric fields on confined water molecules within the pore regions of two representative ion channels, namely potassium and sodium channels. The simulation results reveal that the dynamics of confined water molecules differ significantly from those in the bulk phase, exhibiting pronounced orientation polarization and reduced diffusion rates. These differences arise from the unique geometry and electrostatic environment within the channel pore, which together create a distinct micro-environment.

Further investigation revealed that terahertz electric fields regulate the dynamics of water molecules by exciting vibration modes at specific frequencies. The 16 THz field predominantly excites the rock mode of water molecules. When the field is aligned parallel to the pore axis (*z*-axis), it significantly enhances water molecule motion along the pore axis. In contrast, a perpendicular field suppresses this motion. In contrast, the 24 THz field mainly excites the wag mode and shows an opposite directional dependence in its regulatory effect compared to the 16 THz field. The frequency- and direction-dependent modulation of water molecule behavior can further influence the hydrogen-bonding network and interactions between water molecules and channel protein residues, thereby regulating ion transmembrane transport and affecting the channel function.

This study elucidates the regulatory mechanism of terahertz electric fields on water molecules confined within the pore regions of ion channels, confirming their potential as an effective means to modulate water behavior in confined environments. These findings provide critical theoretical foundations and guidance for the development of novel bio-regulation strategies and the functional control of biomimetic nanofluidic systems.

## Author contributions

Xiaofei Zhao: data curation, validation, investigation, writing—manuscript, methodology, experimental design, visualization. Wen Ding: data curation, validation, methodology. Hongguang Wang: project administration, methodology, writing—review and editing. Yongdong Li: project administration. Chunliang Liu: project administration. All the authors have approved the final manuscript.

## Conflicts of interest

There are no conflicts to declare.

## Supplementary Material

NA-OLF-D5NA00942A-s001

NA-OLF-D5NA00942A-s002

NA-OLF-D5NA00942A-s003

## Data Availability

The structural files used to generate the data presented in this manuscript are publicly available at https://zenodo.org/records/17192687. Supplementary information (SI): additional figures and analyses supporting the main conclusions of this work, including the simulation system setup, water density distributions, structural stability, solvent-accessible pore volumes, and analyses of water dynamics under representative terahertz field frequencies, as well as videos illustrating ion and water motion in the channels. See DOI: https://doi.org/10.1039/d5na00942a.
