# Supplementary material for: Modulation of confined water dynamics in ion channels by terahertz electric fields
Source: Nanoscale Adv. 2026 Feb 2;8(5):1512–21. doi: 10.1039/d5na00942a (PMC12908654; doi:10.1039/d5na00942a)
Supplement: NA-008-D5NA00942A-s001 [file NA-008-D5NA00942A-s001.pdf]

Supplementary Information (SI) for Nanoscale Advances.
This journal is © The Royal Society of Chemistry 2026

Supplemental Information

Modulation of Confined Water Dynamics in Ion Channels by Terahertz Electric Fields

Xiaofei Zhao [1], Wen Ding [1], Hongguang Wang [1]*,
Yongdong Li [1] and Chunliang Liu [1]

[1] *Key Laboratory for Physical Electronics and Devices of the Ministry of Education, School of Electronic Science and Engineering, Xi'an Jiaotong University, Xi'an, Shaanxi 710049, China*

*corresponding authors: wanghg@xjtu.edu.cn

Figure S1 shows the complete molecular dynamics simulation systems used in this study. The protein channels are displayed using the New-cartoon representation, while lipid molecules and water are shown in the Lines representation. Ions are depicted as spheres, with green indicating K^+, blue indicating Na^+, and yellow indicating Cl^-. The simulation box dimensions were 8.69 × 8.69 × 8.73 nm for the Kv1.2 potassium channel system and 8.80 × 8.80 × 12.72 nm for the Nav1.5 sodium channel system, respectively.

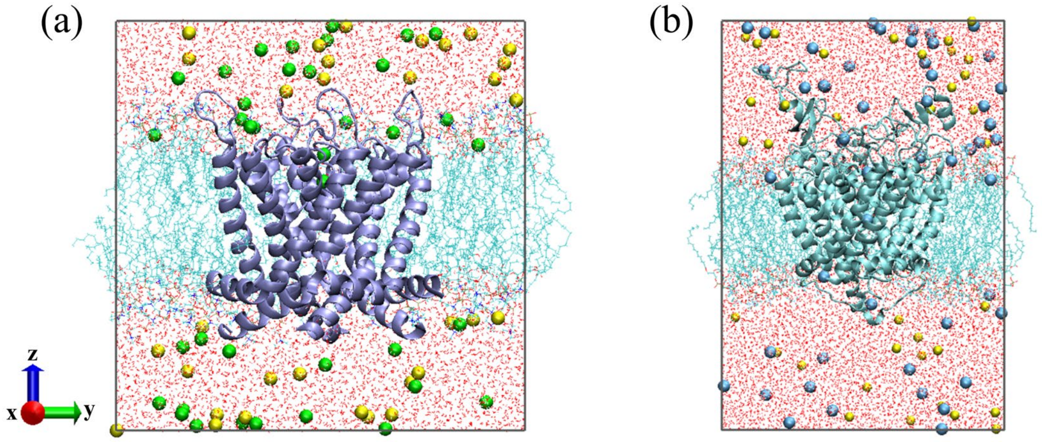

Figure S1: Schematic representation of the molecular dynamics simulation systems. (a) The simulation system of the Kv1.2 potassium channel. (b) The simulation system of the Nav1.5 sodium channel.

The number density distribution of water molecules within the pore is shown in Supplementary **Figure S2**. In the cavity region (approximately 2–4 nm), both channels remain well hydrated. The selectivity filter (SF) of the sodium channel exhibits a hydration level comparable to that of the cavity. In contrast, the SF of the potassium channel (approximately 4.5–6 nm) shows a markedly reduced water density, reflecting the single-file arrangement in which each site accommodates at most one water molecule, or may remain unoccupied.

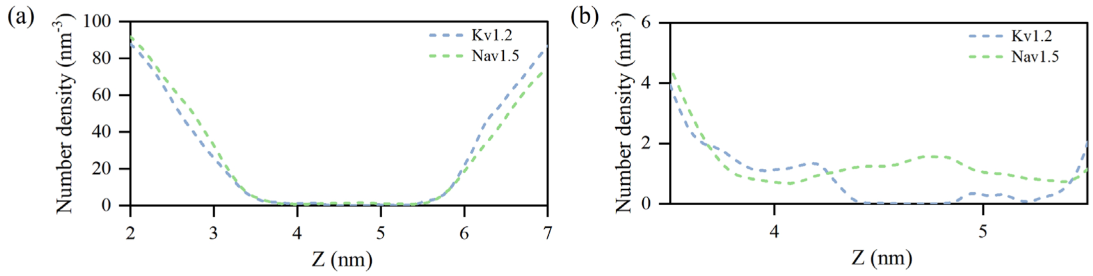

Figure S2: Number density distribution of water molecules. (a) Water molecule number density along the entire pore region. (b) Enlarged view of the number density distribution in the 3–6 nm segment of the pore.

Figure S3 presents the root-mean-square deviations (RMSD) of the channel structures under electric fields of different frequencies, indicating the extent of deviation from their initial conformations during the simulations. Overall, the sodium channel exhibits slightly higher RMSD values than the potassium channel, likely due to its larger extracellular domain and greater structural flexibility. The application of terahertz electric fields does not induce significant changes in the RMSD, suggesting minimal impact on the overall structural stability of the channels.

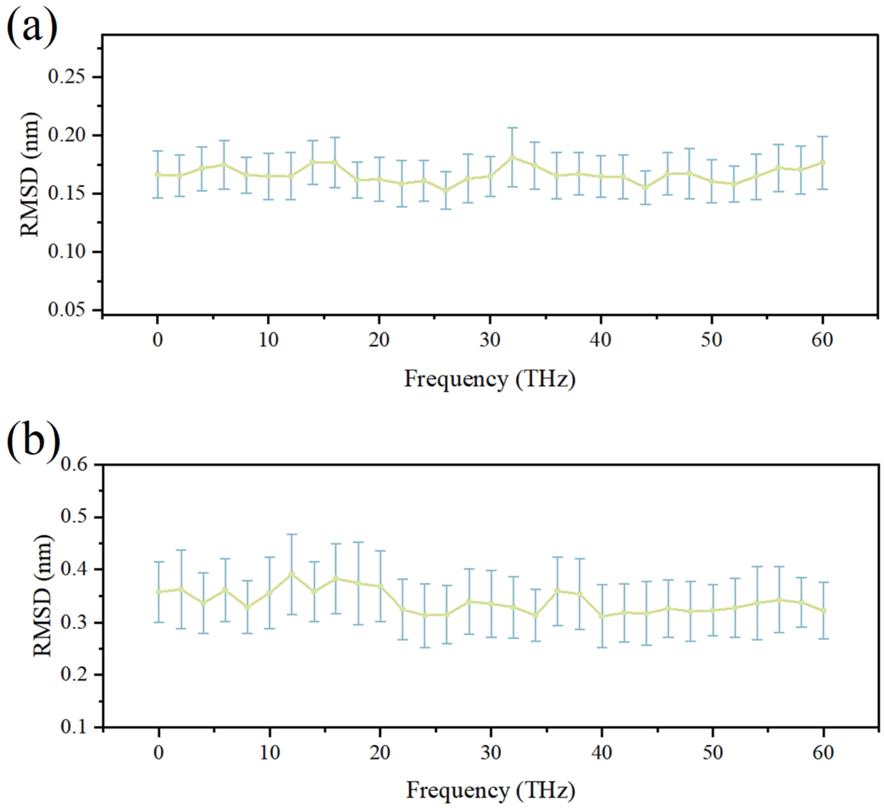

Figure S3: RMSD of ion channels under different electric field conditions. (a) RMSD of the potassium channel. (b) RMSD of the sodium channel.

To assess the potential impact of terahertz electric fields on the channel's capacity to accommodate water molecules, the solvent-accessible volume of the pore region was calculated under different field frequencies, as shown in **Figure S4**. This parameter directly reflects the volume within the pore that can be occupied by water molecules. The results reveal that the solvent-accessible volume remains largely unchanged across all field conditions, suggesting that the channel geometry is structurally stable and not significantly perturbed by the applied terahertz fields. Therefore, the observed alterations in water behavior within the pore are unlikely to result from expansion or constriction of the channel architecture, but rather reflect direct frequency-dependent modulation of confined water dynamics by the external field.

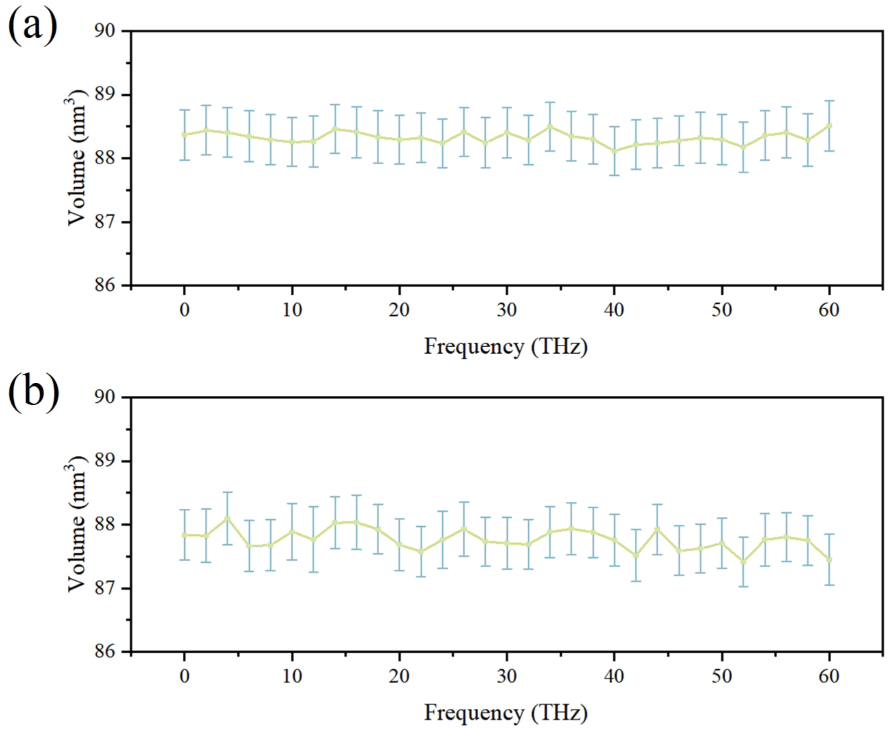

Figure S4: Solvent-accessible volumes within the pore regions. (a) Solvent-accessible volume in the pore region of the potassium channel. (b) Solvent-accessible volume in the pore region of the sodium channel.

Figure S5 presents the z-resolved residence time profiles of water molecules under different electric-field conditions. Comparisons are made between the no-field case and the 16 THz and 24 THz fields, as these two frequencies induce the most pronounced changes in the pore-averaged residence times shown in Fig. 3. Panel (a) corresponds to the potassium channel, and panel (b) to the sodium channel.

The response of water residence times to the applied field varies markedly along the permeation pathway. In the potassium channel, the highly confined SF imposes strong geometric restrictions, which limits the effect of the field in this region. In contrast, the cavity region is strongly modulated by the applied fields, whereas water molecules near the gate exhibit weaker responses due to their proximity to the bulk solution. In the sodium channel, the SF is less confined, allowing the electric field to more effectively modulate water dynamics in both the SF and the cavity. Additionally, an extracellularly confined region in the sodium channel shows substantial field-induced modulation, particularly under the 16 THz field.

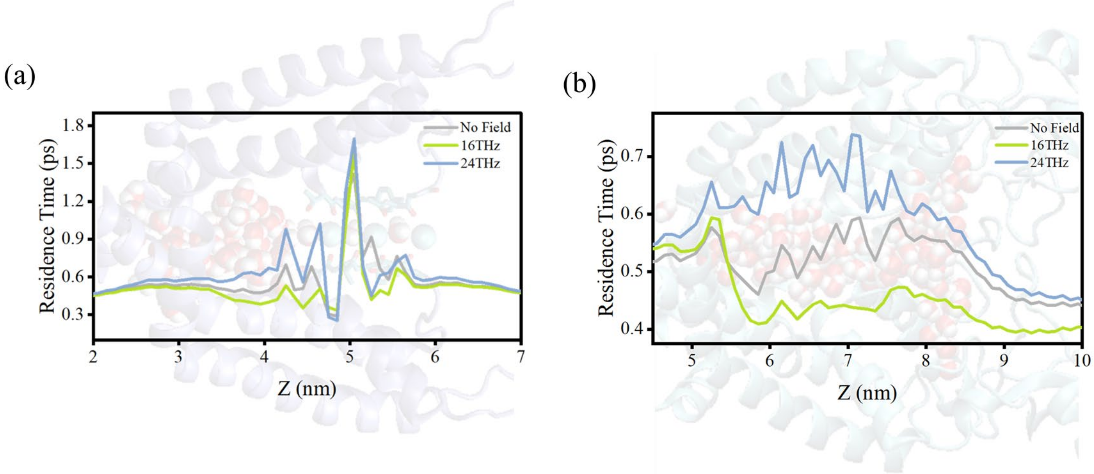

Figure S5: Effect of 16 THz and 24 THz electric fields on the z-resolved residence time profiles of water molecules. (a) Potassium channel. (b) Sodium channel.

Figure S6 presents the distribution of H–H vector components of water molecules in the sodium channel projected onto the x–z plane, illustrating changes in molecular orientation under different field conditions. Under field-free conditions, the distribution is relatively concentrated, indicating limited rotational freedom. When a 16 THz electric field is applied, the distribution becomes more dispersed, suggesting enhanced rotational dynamics consistent with the excitation of the rock librational mode. This increased rotational mobility facilitates reorientation and potentially enhances axial transport. In contrast, the application of a 24 THz field leads to a more focused H–H vector distribution, indicating that the wag librational mode is predominantly excited, thereby restricting rotational motion. These observations reinforce the notion that water molecules exhibit mode-selective responses to terahertz electric fields, resulting in distinct orientational dynamics within confined nanochannels depending on the field frequency.

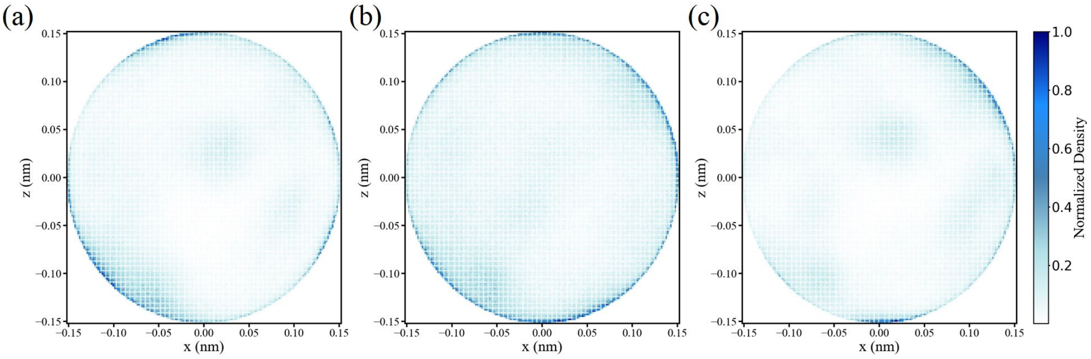

Figure S6: Influence of terahertz electric fields on the rotational orientation of water molecules. (a) Distribution of H–H vectors of water molecules in the sodium channel on the x–z plane under field-free conditions. (b) Distribution of H–H vectors under a 16 THz electric field. (c) Distribution of H–H vectors under a 24 THz electric field.

Figure S7 illustrates the effects of terahertz electric fields on the interactions between water molecules and the sodium channel. Figure S7(a) shows the distribution of hydrogen bonds formed between water molecules and residues within the channel pore under different electric field conditions. The results indicate that under the 16 THz electric field, the number of hydrogen bonds decreases, whereas under the 24 THz field, the average number increases. Specifically, the average number of hydrogen bonds decreases by approximately 17.8% at 16 THz and increases by about 20.8% at 24 THz. These findings suggest that the 16 THz field weakens water－protein interactions, while the 24 THz field strengthens them. Figure S7(b) presents the radial distribution function (RDF) of water molecules in the pore region, calculated with reference to the DEKA motif in the selectivity filter (SF) of the sodium ion channel. It can be observed that water molecules are more loosely distributed under the 16 THz field, whereas they are more tightly packed under the 24 THz field. This supports our previous analysis that the 16 THz field tends to disrupt water–channel binding, while the 24 THz field promotes binding stability.

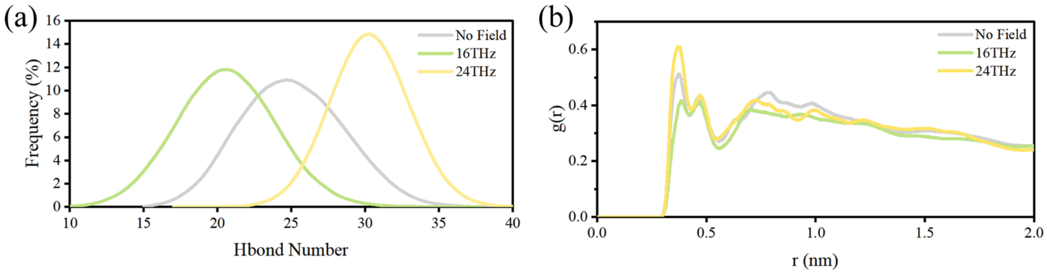

Figure S7: Effects of terahertz electric fields on the binding between water molecules and the sodium ion channel. (a) Distribution of hydrogen bonds formed between water molecules and residues in the pore region of the sodium ion channel; (b) Radial distribution function (RDF) of water molecules within the channel pore under different terahertz electric field conditions.

Figure S8 illustrates the effects of two representative electric field frequencies, 16 THz and 24 THz, on the distribution of water molecules within the confined pore of the potassium channel. Panel (a) shows the change in the number of water molecules within 0.5 nm of each residue under the applied fields relative to the field-free case. The 16 THz field generally reduces the number of water molecules, whereas the 24 THz field increases it, consistent with the trends observed in the radial distribution functions shown in Figure 5b. Panels (b) and (c) depict the local water distribution around residues 399VAL and 402ILE under 16 THz and 24 THz fields, respectively. For clarity, only water molecules within 0.3 nm of the residues are displayed, and representative frames were selected in which the number of water molecules in the region is close to the average. In each panel, the left image corresponds to the field-free condition, and the right image shows the system under the applied field. It is evident that the 16 THz field reduces the number of nearby water molecules and produces a more dispersed distribution, while the 24 THz field increases the local water density and results in a more compact arrangement. These two residues were selected for visualization because, as shown in Figure 5c of the manuscript, they exhibit the largest changes in water–residue interaction energies under the 16 THz and 24 THz fields, respectively.

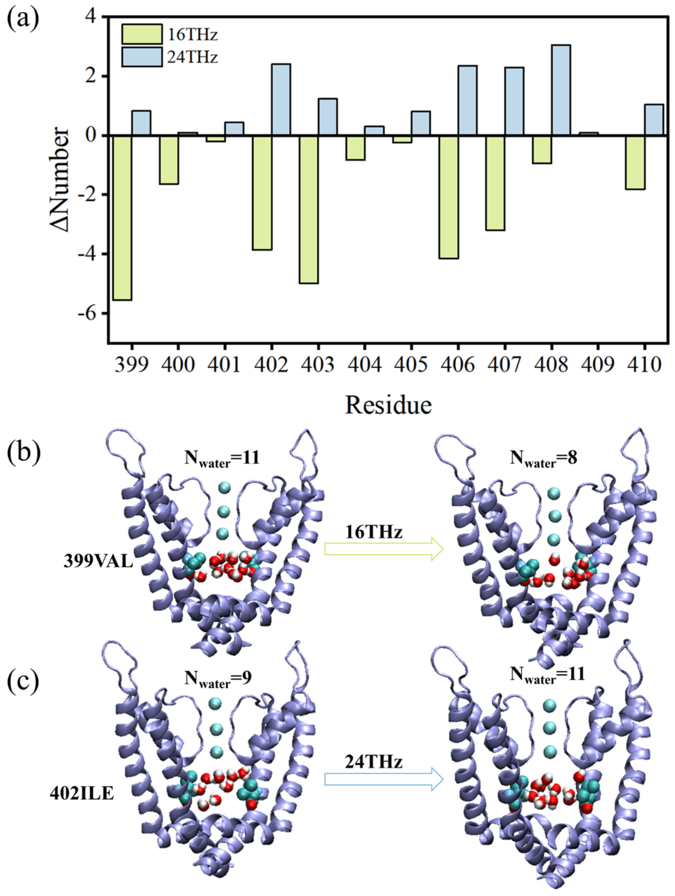

Figure S8: Effects of terahertz electric fields on water distribution within the channel pore. (a) Change in the number of water molecules within 0.5 nm of specific residues under applied fields relative to the field-free condition. (b) Local water distribution within 0.3 nm of residue 399VAL under a 16 THz field. (c) Local water distribution within 0.3 nm of residue 402ILE under a 24 THz field.

Figure S9 illustrates the effects of terahertz electric fields on the interaction energy between water molecules and the sodium ion channel. Due to the presence of four asymmetric subunits in the sodium ion channel, its structure is more complex than that of the potassium channel. For clarity in visualization and description, residues are labeled using Index numbers, and their corresponding residue IDs and residue names are listed in Table S1 for accurate reference. To highlight the differential effects of the 16 THz and 24 THz electric fields on water–channel interactions, only representative residues exhibiting opposite trends in interaction energy changes under the two field conditions are shown. The direction of energy change is indicated by color: yellow dots represent increased interaction energy relative to the field-free condition, while green dots indicate decreased interaction energy.

The results show that under the 16 THz field, the interaction energy between water molecules and channel residues generally increases. In particular, the interaction energy involving Index 2 (138D) increases by approximately 33 kJ/mol, suggesting that this frequency weakens water binding to the channel. In contrast, under the 24 THz field, the interaction energy tends to decrease, with the most significant reduction observed for Index 17 (396E), where the energy decreases by about 35 kJ/mol. This indicates that the 24 THz field facilitates stronger binding between water molecules and the channel.

These findings further support our previous analysis: the 16 THz electric field tends to weaken water–channel interactions, whereas the 24 THz field enhances them.

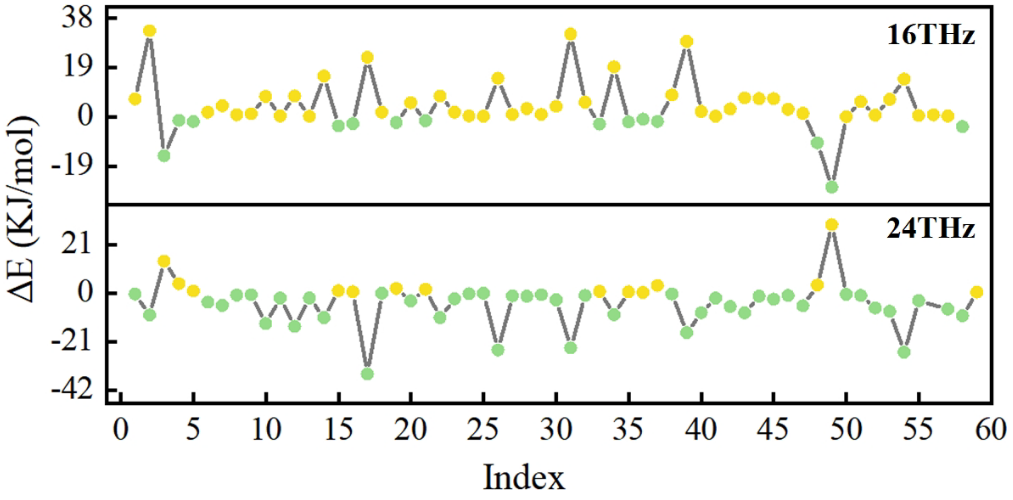

Figure S9: Effect of terahertz electric fields on the interaction energy between water molecules and residues of the sodium ion channel. The correspondence between indices and residue identities is listed in Table S1.

Table S1. Mapping of indices in Figure S9 to residue IDs in the sodium ion channel

Index	Residue	Resname	Index	Residue	Resname
**1**	137	Q	**30**	668	F
**2**	138	D	**31**	669	K
**3**	139	C	**32**	670	G
**4**	143	L	**33**	671	W
**5**	144	Y	**34**	673	D
**6**	147	T	**35**	674	I
**7**	150	S	**36**	675	M
**8**	168	F	**37**	678	A
**9**	169	Y	**38**	679	V
**10**	172	N	**39**	680	D
**11**	174	I	**40**	709	F
**12**	175	L	**41**	711	T
**13**	176	A	**42**	712	L
**14**	392	G	**43**	713	N
**15**	394	W	**44**	715	F
**16**	395	I	**45**	718	V
**17**	396	E	**46**	926	A
**18**	397	T	**47**	927	G
**19**	401	C	**48**	928	W
**20**	402	M	**49**	929	D
**21**	404	V	**50**	932	L
**22**	422	N	**51**	975	F
**23**	423	L	**52**	976	L
**24**	424	V	**53**	979	V
**25**	425	V	**54**	980	N
**26**	427	N	**55**	981	M
**27**	428	L	**56**	983	I
**28**	430	L	**57**	984	A
**29**	431	A	**58**	985	I

Figure S10 shows the exchange rate of water molecules between the confined region and the bulk solution in the sodium ion channel. Using the DEKA residues in the SF as the reference, the retention ratio of water molecules within 1.5 nm was calculated over time. The results indicate that a 16 THz electric field significantly accelerates water exchange, reducing the average residence time by 63.6%. In contrast, a 24 THz field slows down the exchange, increasing the average residence time to 1.42 times that observed under field-free conditions.

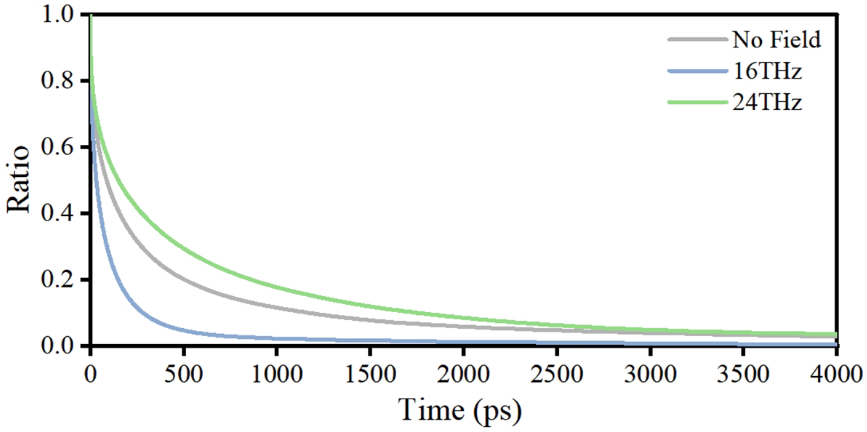

Figure S10: Terahertz field－induced modulation of water exchange dynamics in the confined region of the sodium channel.

Figure S11 illustrates the directional dependence of the regulatory effects of 16 THz and 24 THz electric fields on the dynamics of water molecules within the sodium ion channel. The direction of the electric field is defined by the angle between the field vector and the z-axis. As shown in Figure S11 (a), when the field forms an angle of approximately 60° with the z-axis, the effect of the electric field on water dynamics reverses: the 16 THz field shifts from enhancing to suppressing water motion, whereas the 24 THz field shifts from suppressing to enhancing it. Figures S11 (b) – (c) further present the results for different azimuthal angles within the xy plane (0°, 45°, and 90° relative to the x-axis). It can be observed that the response of water molecules to the terahertz electric field primarily depends on the angle with respect to the z-axis, while remaining largely insensitive to variations in the field direction within the xy plane.

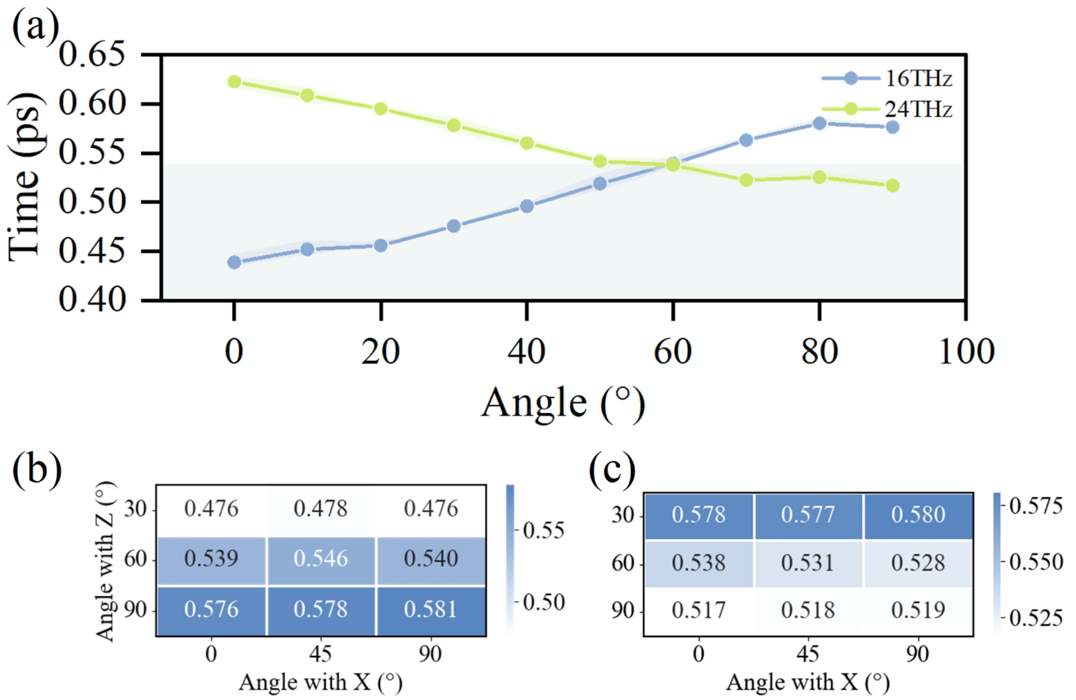

Figure S11: Directional dependence of terahertz electric field effects on water dynamics within the pore region of the sodium channel. (a) Modulation of water molecule motion by electric fields with varying angles relative to the z-axis in the xz plane. (b) Directional dependence of water dynamics under a 16 THz electric field. (c) Directional dependence of water dynamics under a 24 THz electric field.

Figure S12 shows the dependence of the regulatory effect of terahertz electric fields on water molecules within the sodium ion channel on the field strength. The results indicate that when the field strength exceeds approximately 0.1 V/nm, the dynamical behavior of water molecules begins to change noticeably, and further increases with increasing field strength. Notably, the 24 THz electric field applied parallel to the membrane surface exhibits a markedly weaker sensitivity to field strength compared to other conditions.

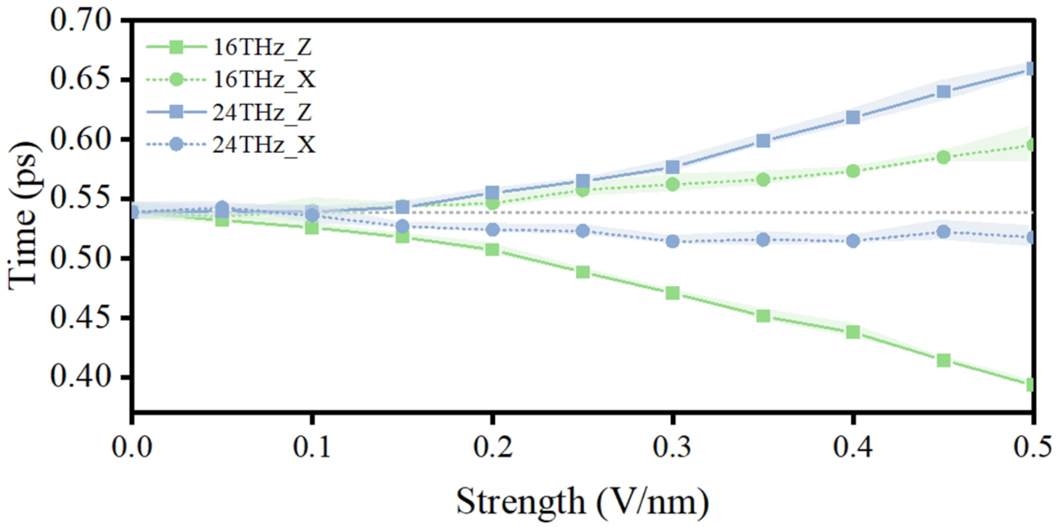

Figure S12: Field-strength dependence of terahertz electric field effects on water molecules in the sodium channel.

Notes on Supplementary Videos 1 and 2:

Supplementary Video S1 illustrates the motion of potassium ions and water molecules in the potassium channel. Spheres of different colors represent distinct potassium ions, while the oxygen atoms of water molecules are color-coded to distinguish different water molecules, and hydrogen atoms are represented by white spheres. In the selectivity filter (SF) of the potassium channel, water molecules and potassium ions are arranged in a single-file manner. The video is displayed with a frame interval of 1 ns, highlighting that water molecules in the SF cannot freely exchange, consistent with the pronounced orientational restriction and slowed motion observed in Figure 2.

Supplementary Video S2 depicts the motion of water molecules in the sodium channel. The oxygen atoms of the four water molecules closest to the DEKA residue sequence in the SF are color-coded to differentiate them, while the hydrogen atoms are represented by white spheres. Other water molecules are shown in licorice representation, with white for hydrogen atoms and red for oxygen atoms. The SF of the sodium channel is wider than that of the potassium channel, allowing multiple water molecules to occupy the same z position. With a frame interval of 1 ns, the video demonstrates that water molecules in the SF can move relatively freely and exchange with water molecules in the extracellular and cavity regions. As shown in Figure 2, the orientational restriction and slowed motion of water molecules in the sodium channel are considerably weaker than in the potassium channel.
